# The Genome of the Korean Island-Originated *Perilla citriodora* ‘Jeju17’ Sheds Light on Its Environmental Adaptation and Fatty Acid and Lipid Production Pathways

**DOI:** 10.3390/genes14101898

**Published:** 2023-09-30

**Authors:** Seon-Hwa Bae, Myoung Hee Lee, Jeong-Hee Lee, Yeisoo Yu, Jundae Lee, Tae-Ho Kim

**Affiliations:** 1 Genomics Division, Department of Agricultural Biotechnology, National Institute of Agricultural Sciences, Rural Development Administration, Jeonju 54874, Republic of Korea; bae209@korea.kr; 2 Upland Crop Breeding Research Division, Department of Southern Area Crop Science, Rural Development Administration (RDA), Miryang 50424, Republic of Korea; emhee@korea.kr; 3 SEEDERS Inc., 118, Jungang-ro, Jung-gu, Daejeon 34912, Republic of Korea; jhlee@seeders.co.kr; 4 DNACARE Co., Ltd., 48, Teheran-ro 25-gil, Gangnam-gu, Seoul 06126, Republic of Korea; yeisooyu@dnacare.co.kr; 5 Department of Horticulture, College of Agriculture and Life Sciences, Jeonbuk National University, Jeonju 54896, Republic of Korea

**Keywords:** *Perilla citriodora*, fatty acid biosynthesis, adaptation, triacylglycerol biosynthesis, transcriptomics, fatty acid desaturase

## Abstract

*Perilla* is a key component of Korean food. It contains several plant-specialized metabolites that provide medical benefits. In response to an increased interest in healthy supplement food from the public, people are focusing on the properties of Perilla. Nevertheless, unlike rice and soybeans, there are few studies based on molecular genetics on Perilla, so it is difficult to systematically study the molecular breed. The wild *Perilla*, *Perilla citriodora* ‘Jeju17’, was identified a decade ago on the Korean island of Jeju. Using short-reads, long-reads, and Hi-C, a chromosome-scale genome spanning 676 Mbp, with high contiguity, was assembled. Aligning the ‘Jeju17’ genome to the ‘PC002’ Chinese species revealed significant collinearity with respect to the total length. A total of 31,769 coding sequences were predicted, among which 3331 were ‘Jeju17’-specific. Gene enrichment of the species-specific gene repertoire highlighted environment adaptation, fatty acid metabolism, and plant-specialized metabolite biosynthesis. Using a homology-based approach, genes involved in fatty acid and lipid triacylglycerol biosynthesis were identified. A total of 22 fatty acid desaturases were found and comprehensively characterized. Expression of the FAD genes in ‘Jeju17’ was examined at the seed level, and hormone signaling factors were identified. The results showed that the expression of FAD genes in ‘Jeju17’ at the seed level was high 25 days after flowering, and their responses of hormones and stress were mainly associated with hormone signal transduction and abiotic stress via cis-elements patterns. This study presents a chromosome-level genome assembly of *P. citriodora* ‘Jeju17’, the first wild *Perilla* to be sequenced from the Korean island of Jeju. The analyses provided can be useful in designing ALA-enhanced Perilla genotypes in the future.

## 1. Introduction

*Perilla* is a member of the mint family and is distributed mainly in East Asia, including China, Japan, and South Korea [1]. The plant is traditionally used as a fragrance and herbal medicine [2]. It is also employed in traditional food as a leafy vegetable for wrapping boiled rice or meat [2]. *Perilla* is also employed as a complement for poultry [3] and livestock feed [4,5,6].

To the best of our knowledge, the *Perilla* genus encompasses one tetraploid species (cultivated) named *Perilla frutescens* and two diploid species (wild), *Perilla citriodora* and *Perilla setoyensis* [1,7]. Although *Perilla* is well distributed in East Asia, China is assumed to be the primary center of diversity [2,8]. 

In the last decade, *Perilla* has captured the attention of scientists due to its rich diversity of phytochemical compounds, including volatile oils, flavonoids, triterpenes, tocopherols, phytosterols, polycosanols, quinines, steroids, alkaloids, fatty acids, and others [8,9]. Therefore, *Perilla* presents diverse health-promoting benefits such as anti-cancer [10,11,12], anti-inflammatory [13,14], anti-cough [15], antioxidant [16,17,18], anti-depressive [19,20,21,22], and anti-allergic [23,24] properties. Perilla oil has a unique fatty acid composition that is high (50–60%) in vegetable omega-3 polyunsaturated fatty acid (PUFA, about 70%) and a-linolenic acid (ALA, C18:3, ω−3, about 60–68%) [25]. The major fatty acids of Perilla oil are linolenic acid (LA, C18:3), linoleic acid (C18:2, ω−6), and oleic acids (OA, C18:1, ω−9) [26]. Perilla is an important oilseed crop with the highest ALA level [27]. It has also been reported that neutral lipids (more than 90%) are a major lipid class in Perilla oil, of which about 90% are triacylglycerols [26]. Moreover, neutral lipids (more than 90%) are a major lipid class in Perilla oil, of which about 90% are triacylglycerols [26].

The fatty acid desaturases (FAD) mainly responsible for the biosynthesis from ALA in seed storage lipids are FAD2 and f FAD3. The plastidial delta-12 (∆12), FAD6, plays a key role in linoleic acid and α-linolenic acid biosynthesis [27]. FAD6 genes have been known to be characterized in a variety of plant species, including oil palm [28]. PfFAD6 was expressed in various organs at different levels and responded to multiple biotic and abiotic stresses [27]. As the benefits of Perilla oil for human health have become obvious, there has been an increase in the production of Perilla [29].

Recently, the genome of the tetraploid *P. frutescens* isolate PF40 and the diploid progenitor *P. citriodora* have been released [7]. Both sequenced genomes originated from China and enabled the identification of genes involved in fatty acid biosynthesis and leaf coloring [7]. So far, several fatty acid desaturases have been cloned, and transcriptome investigation has helped to identify key enzymes in the *Perilla* fatty acid and lipid production pathways [30].

In 2002, *P. citriodora* was reported for the first time on a Korean island called Jeju [31]. Jeju Island is a detached territory of Korea, characterized by a humid subtropical climate. The island arose from a volcanic eruption that occurred about 2 million years ago (MYA) [32]. Although the genome of *P. citriodora* has been sequenced, generating high-quality genomic data using diverse genetic resources will help to capture the genetic diversity among *Perilla* species. Therefore, the investigation of particular isolates, especially from the islands, offers an opportunity to explore their species-specific gene repertoire.

The present study aimed to provide high-quality genomic data from *P. citriodora* ‘Jeju17’ in order to determine the genes related to island adaptation as well as the identification of fatty acid and lipid biosynthesis genes.

## 2. Materials and Methods

### 2.1. Whole-Genome Sequencing, Genome Size Estimation, and Genome Assembly

**Figure 1:**
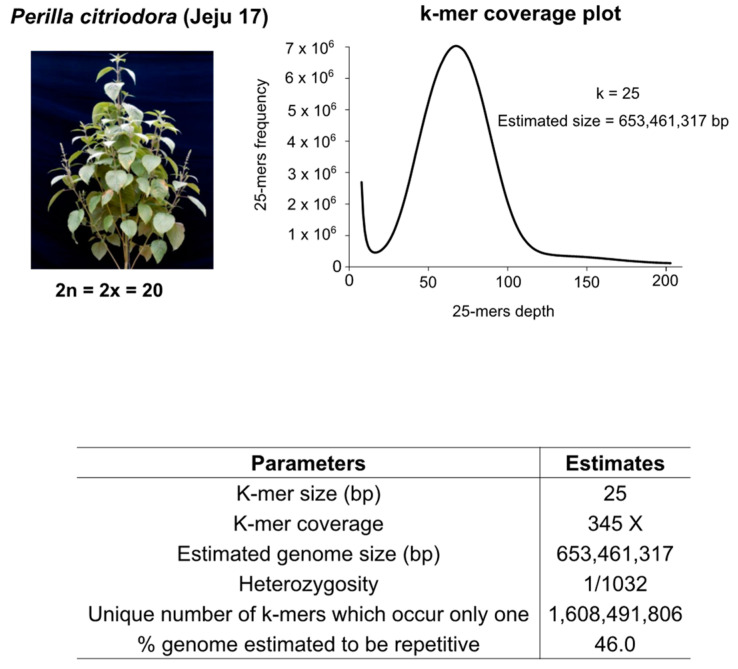
Genome characteristics for *Perilla citriodora* ‘Jeju17’.

*P. citriodora* ‘Jeju17’ (Figure 1) was discovered and collected on Jeju island (33°57′24.2424″ N 84°7′52.77″ W) in 2002 and preserved at the National Institute of Crops Science of Korea [31]. It was used as a material by advancing more than 10 generations advancement. Two-week-old leaves were sampled for whole-genome sequencing using Illumina, PacBioRSII, and Hi-C platforms (Figure S1). A genome survey was implemented to estimate the plant genome size using the Jellyfish v2.2.7 tool [33]. For annotation purposes, short-read RNA sequencing was performed on seed, bud, and leaf tissues, following the manufacturer’s protocols. The sequence reads archive (SRA) for the genome and transcriptome data are provided in Table S1. 

A hybrid assembly strategy (Figure S1) combining short and long reads was conducted to produce a chromosome-scale assembly. Briefly, after an initial assembly with Platanus v1.2.1 [34], gaps were filled using PBJELLY v2.0 [35], and the redundant sequences were filtered out with CD-HIT [36]. Meanwhile, long-read assemblies were executed with Falcon v0.7.0 [37]. Both assemblies were merged using HaploMerger v2 [38]. The hybrid assembly was shipped to Phase Genomics^®^ for the Hi-C scaffolding step.

### 2.2. Genome Assembly Quality Assessment

The assembly was assessed for contiguity, gene completeness, and structural variation by comparison with the reference genome *P. citriodora* PC002 [7]. The chromosomes were arranged from 1 to 10 in order of length (bp). BUSCO analysis [39] was completed using the Embryophyta odb_10 database. Whole-genome alignment with PC002 was performed using MashMap v2.0 [40] and visualized with D-Genies v1.4.0 [41]. In addition, MUMmer v4.0.0 [42] and Assemblytics [43] assessed the structural variants between ‘PC002’ and ‘Jeju17’.

In order to validate the assembled genome, bacterial artificial chromosome (BAC) libraries were produced. High-molecular-weight DNA was extracted from young leaves of ‘Jeju17’, as described by Kang et al. [44]. After a digestion step with *Hin*dIII- and *Bam*HI-restriction enzymes, insert size selection was performed, followed by ligation using a pSMART BAC vector. The ligated substrate was then transformed into DH10B-competent cells. A total of 10 BAC clones were subsequently sequenced using an ABI 3730 × l DNA Analyzer and 454 Life Sciences GS FLX System (GS FLX). BAC clone sequencing was used to select 10 genes (Tables S3 and S4) related to triacylglycerol (TAG) biosynthetic pathways [45]. Assembly was carried out with Newbler v2.8 (https://www.ncbi.nlm.nih.gov/assembly/GCF_000588835.1/, accessed on 24 September 2013). Gap filling was conducted following the primers walking method [46]. The assembly was error-corrected with short-read data. The generated BAC-based assembly was aligned onto the chromosome-scale assembled genome using MUMmer v4.0.0 [42].

### 2.3. Repeat Identification and Genome Annotation

For repeat identification, RepeatModeler v. 1.0.8 (http://www.repeatmasker.org/RepeatModeler/, accessed on 5 February 2014) and RepeatMasker v. 4.0.5 (http://www.repeatmasker.org, accessed on 5 February 2014) packages were employed. Non-coding RNA (rRNA, tRNA, miRNA, and snRNA) was detected using Infernal v1.1.4 [47] with the Rfam database [48] (https://rfam.xfam.org/, accessed on 14 March 2022).

Prior to structural annotation, a genome-based transcript assembly was generated. Raw RNA-seq data were checked and trimmed with FastQC v0.11.2 [49] and Trimmomatic v0.36 [50]. The reads were mapped onto the genome using HISAT v2.2.1 [51]. Then, StrigTie v2.2.1 [52] was used to construct the transcript assembly. Gene prediction was carried out with MAKER2 pipeline [53], while functional annotation was executed by BLASTp [54], HMMER [55], and BLAST2GO [56] searches against plant reference sequences and NCBI non-redundant (nr), UniProt, Pfam, GO, KEGG, and InterProScan databases.

### 2.4. Gene Family Analysis, Time Divergence Estimation, and Phylogenetic Placement of Jeju 17

This study used OrthoFinder v.2.3.12 [57] to determine the conserved and species-specific gene families. Gene sets from Lamiales, including *P. citriodora* ‘Jeju17’, *P. citriodora* ‘PC002’, *P. citriodora* ‘PC099’, *P. frutescens* ‘PF40’, *Salvia splendens*, *Salvia miltiorrhiza*, *Mimulus guttatus*, *Sesamum indicum* var. Zhongzhi13, *Sesamum indicum* var. Goenbaek, and *Olea europeae* were used with *Solanum lycopersicum* and *Arabidopsis thaliana* as the outgroup species (Table S2). 

KEGG and GO enrichment analyses of species-specific gene families were assessed with the KOBAS-I online server [58] using the default settings (statistical test: “Fisher’s exact test”; FDR correction method: “Benjamini and Hochberg”). 

Using the single-copy orthologues, a multiple-sequence alignment (MSA) was performed by MAFFT v.7.464-0 [59]. After trimming the MSA file with trimAl v.1.4.1 [60], a maximum likelihood phylogenetic construction with 1000 replicates was conducted following the JTT+F+R3 model, using IQ-TREE v1.6.12 [61]. 

Time divergence was estimated using the Reltime method [62,63] and the JTT matrix-based model [64]. The calibrations were set to 25.0–57.1 MYA (*Mimulus* versus *Sesamum* genera) and 112.4–125.0 MYA (*Arabidopsis* vs. *Lycopersicum* genera) following the TimeTree database (www.timetree.org, accessed on 6 April 2017).

### 2.5. Identification of Putative Orthologs 

Orthologs among the 12 species were identified by conducting an OthoFinder analysis [52]. Orthologous gene pairs were retrieved from otho groups containing one gene per species. The analysis was conducted as previously described [65,66]. For each ortholog or paralog, the protein sequences were aligned using ClustalW [67], and the corresponding codons were aligned using PAL2NAL [68] (Suyama et al. 2006) with the guidance of coding sequences. The Ka and Ks values were calculated using the Nei Gojobori method [69] implemented in the PAML package [70]. 

### 2.6. Identification of Genes Involved in Fatty Acid and Lipid Triacylglycerol Biosynthesis 

Using the *A. thaliana* acyl-lipid metabolism database [71], we inferred genes involved in fatty acid and triacylglycerol biosynthesis in *P. citriodora* ‘Jeju17’ by performing a protein BLASTp search (E = 1× 10^−10^). A cross-check validation was also performed using the reference genome PC002 from Zhang et al. [7].

Furthermore, using the curated fatty acid desaturase hmm profiles PF00487 and PF03405 (from http://pfam.xfam.org/, accessed on 1 January 2004) in addition to previously identified desaturases from *P. frutescens ‘*PF40’ [72], this study identified and classified fatty acid desaturases that were potentially involved in fatty acid and lipid triacylglycerol biosynthesis in the wild species *P. citriodora* ‘Jeju17’. The study employed both HMMER and BLASTp searches for the identification of candidate genes. The protein domain was checked using PfamScan v1.6 [73]. 

For phylogenetic tree construction, fatty acid desaturases from *P. frutescens* and *S. indicum* served as baits. The study involved a blast search against the *P. citriodora* ‘PC002’ genome and retrieved corresponding desaturase genes that were also included as baits. The sequences were aligned using MAFFT v.7.464-0 [59]. The resulting MSA was trimmed with trimAl v.1.4.1 [60], and the tree was produced with IQ-TREE v1.6.12 [61] using 1000 replicates.

The subcellular localization and chemical properties of the proteins were assessed using ExPASy (http://web.expasy.org/protparam/, accessed on 5 February 2014) and Cell-PLoc (http://www.csbio.sjtu.edu.cn/bioinf/plant/, accessed on 5 February 2014) [74,75]. 

A 2Kbp upstream promoter region was extracted to investigate cis-acting regulatory elements using the PlantCARE webserver (http://bioinformatics.psb.ugent.be/webtools/plantcare/html/, accessed on 5 February 2014) [76].

### 2.7. Synteny Analysis

Gene-to-gene synteny analysis was executed with MCScanX [77] embedded in TBTools [78] using the default settings.

## 3. Results and Discussion

### 3.1. Genome Survey, Sequencing, and Assembly Quality

With initial short-read sequencing data representing 345X coverage, the k-mer-based genome size was estimated as 653 Mbp (Figure 1). By using short- and long-read sequencing platforms, the study generated 339X (Illumina), 1195X (Illumina), 72X (PacBio), and 26X (Hi-C) fold on MiSeq, HiSeq, PacBioRSII, and Hi-C systems, respectively (Figure S1, Table S1). Due to the low quality of the long-read data, the long-read assembly resulted in an N50 of 0.6 Mbp. To address this issue, we opted for hybrid assembly combining both short- and long reads. Therefore, an initial assembly base consisting of diverse insert sizes (Table S1) produced an assembly of 718 Mbp with an N50 of 7.5 Mbp. Redundant sequences were removed, leading to an assembly spanning 644 Mbp with an N50 value of 9.6 Mbp. Furthermore, HaploMerger helped to combine both long- and short-read assemblies to reach an assembly size of 678 Mbp with an N50 of 12.3 Mbp (Figure S1). By adding Hi-C data, the assembly contiguity was drastically improved up to 68 Mbp (N50) for an assembly size of 676 Mbp (Figure S1).

To validate our assembly, BAC alignment identity > 99% was retained (Tables S3 and S4). In addition, BUSCO analysis showed similar single-copy orthologue coverage for assembly (~93%). However, the BUSCO analysis on annotation data revealed higher coverage (84.1%) of ‘Jeju17’ compared to ‘PC002’ (79.8%) (Table S5). 

The assembly results were compared with the already published PC002, which is karyotypic, and ‘Jeju17’, like ‘PC002’, consists of a diploid species (2n  =  2x  =  20). The alignment of ‘Jeju17’ onto ‘PC002’ revealed extensive exact matches (73.78%) (Figure S2). However, inverted regions were noted within chromosomes 1, 3, 4, 6, 7, and 10. In-depth analysis revealed that the structural variants represented 19.1 Mbp of encompassing insertion (3.36 Mbp), deletion (2.57 Mbp), tandem expansion (2.42 Mbp), tandem contraction (0.59 Mbp), repeat expansion (4.59 Mbp), and repeat contraction (5.57 Mbp) (Table S6).

### 3.2. Genome Annotation Features

Prior to gene prediction, repeats and non-coding RNA were assessed. The transposable elements (TE) content of Jeju17 was around 63.37% and mainly dominated by long terminal repeat (LTR) retrotransposon elements (27.39%) (Table S7). In PC002, the TE content was lower (56.7%), with LTR elements estimated at around 22% [7].

The non-coding RNA spanned 416,914 bp, representing 0.001% of the genome. The tRNA (n = 933), rRNA (n = 434), miRNA (n = 127), and snRNA (n = 190) occupied 68 Kbp, 313 Kbp, 16 Kbp, and 19 Kbp, respectively (Table S8). 

A total number of 32,769 coding-protein sequences were predicted (Table S9). Compared to ‘PC002’, ‘Jeju17’ exhibited a higher number of genes (31,273) with a mean mRNA length of 4140 bp (Table S9). 

**Figure 2:**
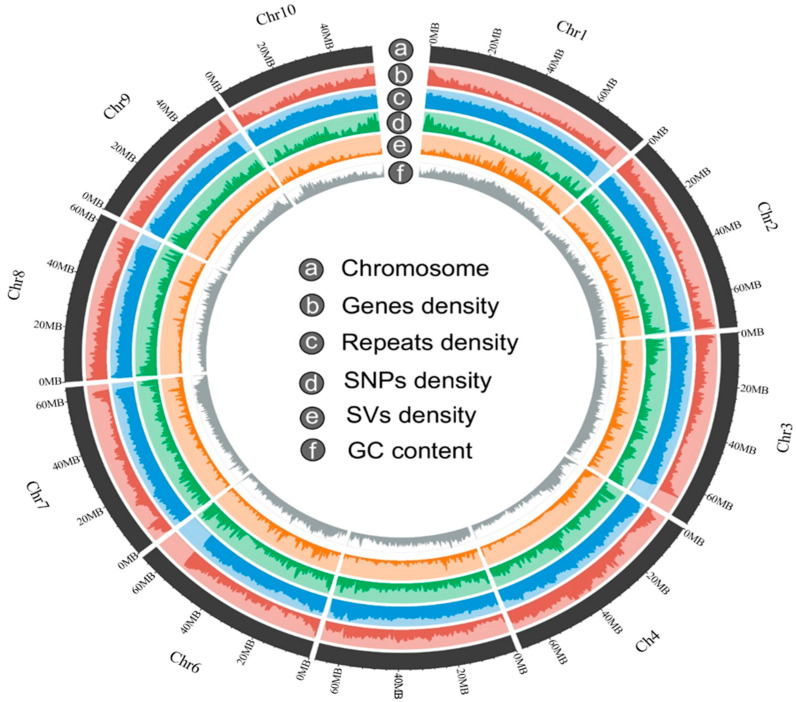
Circos plot of the ‘Jeju17’ genome showing the chromosome (a), genes density (b), repeat density (c), SNPs density (d), structural variant density (e), and GC content (f). The chromosomes are arranged in order of length.

Figure 2 presents the genome map of ‘Jeju17’. 

### 3.3. Genome Evolution

Timetree calculations were retrieved from the TimeTree database and are as follows: *S. lycopersicum* and O. europaea, ranging 75 MYA~88 MYA; O. europaea and U. gibba, ranging 60 MYA~77 MYA; U. gibba and A. majus, ranging 48 MYA~88 MYA; and A. majus and *S. indicum*, ranging 52 MYA~67 MYA. The minimum and maximum branch time estimated for 52 MYA to 67 MYA were applied [79]. In the literature, the estimated time of divergence is 12 MYA for Oleaceae [80] and 62 MYA for Plantaginaceae [81], and the branching time is considered to be consistent with the estimation of the branching time from 11 species. Thus, diploid Perilla was estimated to branch at about 30 MYA. For branch analysis, the Ks value of each gene pair between ‘Jeju17’ and each species was measured and plotted (Figure S3).

### 3.4. Phylogenetic Placement of ‘Jeju17’-Specific Gene Family Assessment

**Figure 3:**
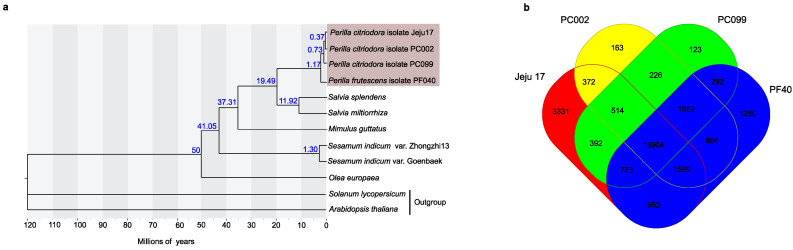
Comparative genomics showing the phylogenetic placement of ‘Jeju17’. (**a**) The phylogenetic tree and divergence times for the other species. The age of divergence is indicated for selected nodes. (**b**) A Venn diagram showing the unique and overlapping gene families in *P. citriodora* ‘Jeju17’, *P. citriodora* ‘PC002’, and *P. citriodora* ‘PF40’.

Using the whole-genome gene sets of 12 species, we inferred the phylogenetic placement of ‘Jeju17’ in the Lamiales order (Figure 3a). As expected, ‘Jeju17’ falls into *Perilla’s* clade and is closely related to *P. citriodora* ‘PC002’. The time-divergence analysis revealed that *Perilla* split from the common ancestor, a member of the Salvia taxa, 1.17 MYA. A comparative analysis of gene orthology within *Perilla* representatives (Figure 3b) showed a set of 123, 163, 1250, and 3331 species-specific gene families for ‘PC099’, ‘PC002’, ‘PF40’, and ‘Jeju17’, respectively.

**Figure 4:**
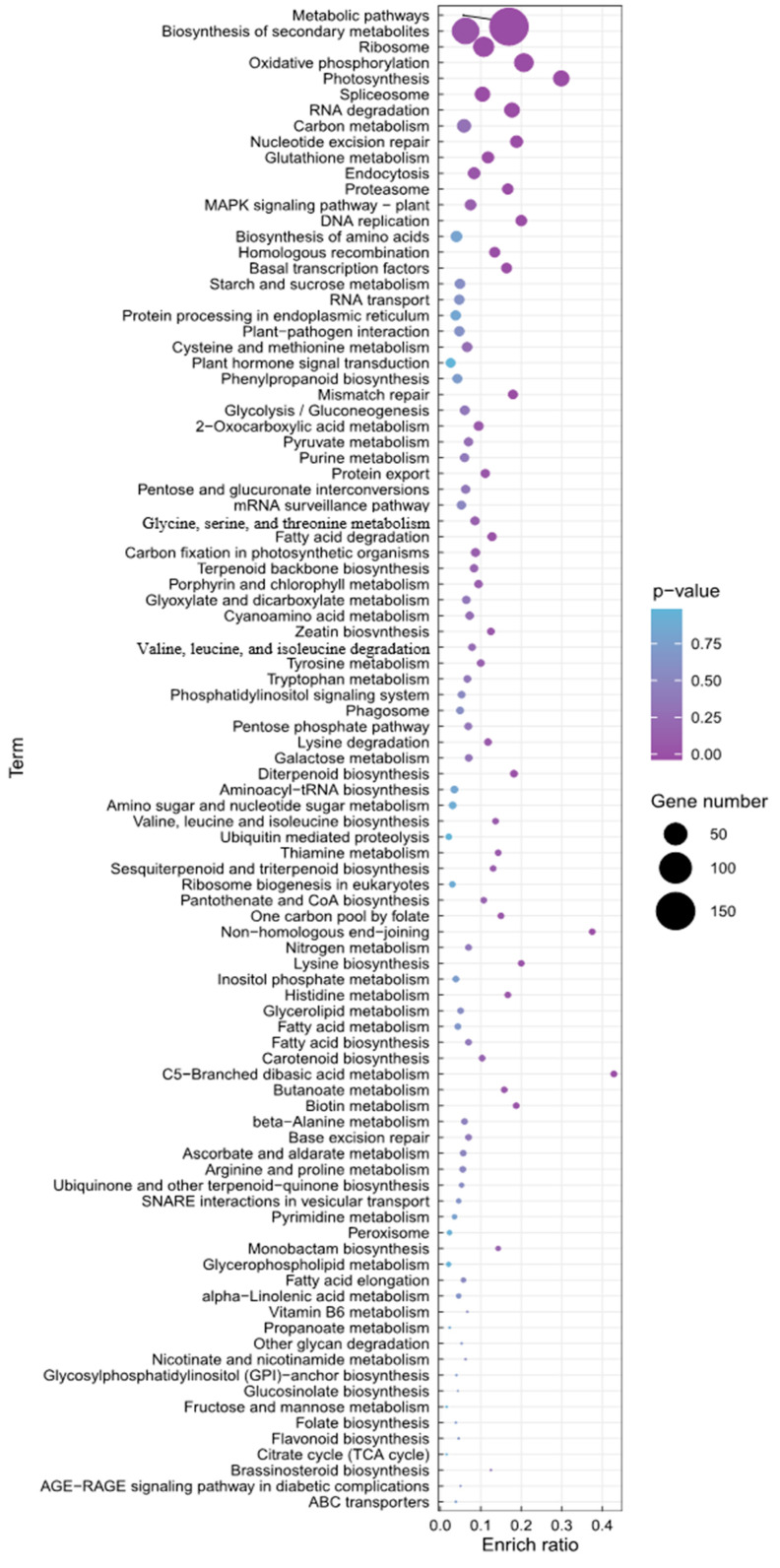
KEGG enrichment bubble plot depicting the functional attributes of the ‘Jeju17’ species-specific gene sets. These differentially expressed genes were grouped into gene pathways using pathway enrichment analysis with the KEGG database. Low q-values are in purple, and high q-values are in blue. The size of the circle is proportional to the number of enriched genes.

To understand the biological implication of species-specific genes, KEGG and GO enrichment analyses were performed (Figure 4, Figures S3, and S4, and Table S10).

The top 100 significantly differential pathways were shown independently in the histogram (Figure S4) and bubble plot (Figure 4).

A KEGG enrichment of ‘Jeju17’ species-specific genes showed diverse biological functions that are mainly related to the biosynthesis of secondary metabolites (ko01100, ko01110) such as sesquiterpenoid and triterpenoid biosynthesis (ko00909), diterpenoid biosynthesis (ko00904), and zeatin biosynthesis (ko00908) (Figure 4, Table S10). As expected, fatty-acid-metabolism-related genes were also identified (ko01230, ko01212, ko00592, ko00062, ko00061, ko00460, ko01210, ko00071). Similar findings were also found in ‘PC002’, ‘PC099’, and ‘PF40’ (Figure S4, Tables S11–S13).

Interestingly, and probably due to the Jeju island subtropical climate, genes related to photosynthesis (ko00195) and adverse environmental responses such as oxidative phosphorylation (ko00190) were detected in the ‘Jeju17’-specific gene set. In addition, genes involved in the plant abiotic stress response, including the MAPK signaling pathway (ko04016), ubiquitin–proteasome system (ko03050), and nucleotide excision repair (ko03420), were identified.

The MAPK machinery pathway is well known for its role in the transfer of information from sensors to initiate cellular responses in environmental stress conditions [82,83,84]. To protect the plant against DNA damage caused by stresses such as exposure to ultraviolet light, nucleotide excision repair can be adopted by the plant to mitigate the damage caused by DNA lesions and therefore maintain genome integrity [85]. Furthermore, the ubiquitin–proteasome system is also deployed in adverse growth conditions to facilitate cellular changes by regulating the abundance of regulatory and structural proteins and enzymes [86,87]. Overall, ‘Jeju17’ harbored not only fatty acid and specialized metabolite genes but also the genetic resources to tolerate environmental stress such as salt stress, high light, temperature, and heat. These results suggest that the lipid biosynthetic pathway is related to the physiological and environmental conditions of plants.

To compare ‘Jeju17’ and ‘PC002’, KEGG analysis was performed using the Perilla sequence reported in China (Figure 4 and Figure S4A). As a result, the percentage of gene annotations related to metabolic pathways was high in ‘PC002’, and the analysis result was different from ‘Jeju17’ (Figure S4A).

### 3.5. Identification of Genes Involved in Fatty Acid and Lipid Triacylglycerol Biosynthesis

**Figure 5:**
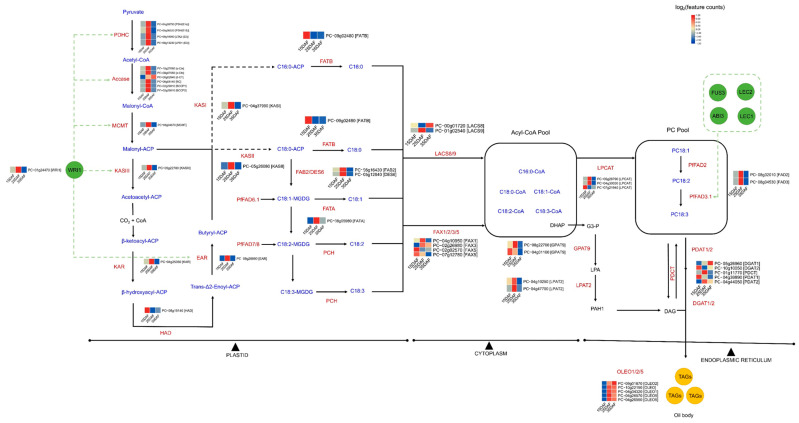
A simplified overview of the fatty acid and lipid triacylglycerol biosynthesis pathways in *Perilla*. The heat map shows transcript changes in triacylglycerol biosynthesis. Upregulated genes are in red, and downregulated genes are in green.

A comparative analysis of genes involved in fatty acid and lipid triacylglycerol biosynthesis revealed consistent gene conservation between ‘PC002’ and ‘Jeju17’ (Figure 3c). Based on the identified genes, we reconstructed the fatty acid and lipid triacylglycerol biosynthesis pathways (Figure 5). As reviewed by Bae et al. [25], palmitic acid (C16:0), stearic acid (C18:0), and oleic acid (C18:1) were produced in plastids and transported into the cytoplasm, where they entered an acyl-CoA pool for esterification at the sn-2 position, generating phosphatidylcholine by the acyl-CoA:lysophosphatidylcholine acyltransferase (LPCAT) enzyme (PC_05g28790, PC_04g30030, and PC_07g21940) (Figure 5). After desaturation in the endoplasmic reticulum, oleic acid is transformed into linoleic acid and alpha-linolenic acid by FAD2 (PC_08g32010) and FAD3 (PC_08g04530) catalysts. The derived fatty acids were then transacylated or sent to the acyl-CoA pool by LPCAT for insertion into triacylglycerol production through the Kennedy pathway (Figure 5). The transcription factor WR1, known to actively regulate fatty acid biosynthesis [88,89], was also identified in the ‘Jeju17’ genome, paving the way for functional validation and potential fatty acid and/or lipid bioengineering from wild *Perilla*. The mechanism of the regulation of FA synthesis by TFs in Perilla is still unknown. Meanwhile, Moreno-Perez et al. suggested the implications of histone methylation (H3K4me3) for fatty acid biosynthesis and interaction with TFs in sunflowers. Moreover, acetyl-CoA, an organelle agent involved in fatty acid synthesis in plants, has been found to correlate with histone acetylation and DNA methylation in *A. thaliana* through a beta-oxidation process [90]. Therefore, the investigation of the epigenome of Perilla and identified TFs, such as ABI3, FUS3, LEC1, and LEC2, will open up new avenues to decipher the entire landscape of fatty acid biosynthesis in Perilla.

**Figure 6:**
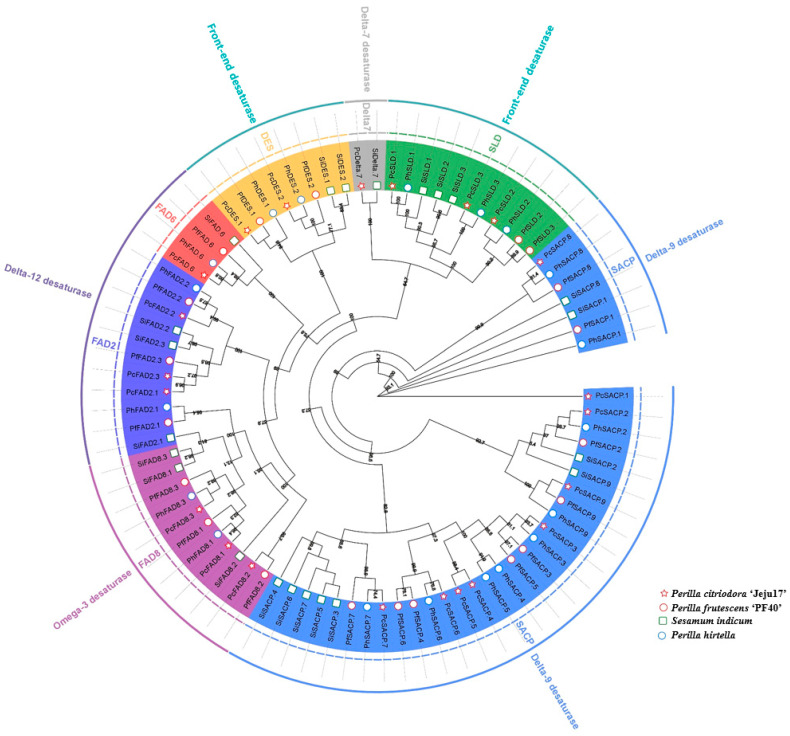
Phylogenetic tree of fatty acid desaturases (FAD) genes with ‘Jeju17’, ‘PC002’, *Perilla frutescens* ‘PF40’, and *Sesamum indicum* genes as baits. Each subfamily group of FAD gene family members has a differently colored label; there are five FAD subfamilies.

This study also explored the identification of fatty acid desaturases, which play key roles in fatty acid production. A total of 22 fatty acid desaturases (Figure 6, Table S14) were identified and grouped into 5 families, including delta-12 desaturase (n = 4), front-end desaturase (n = 5), delta-7 desaturase (n = 1), delta-9 desaturase (n = 9), and omega-3 desaturase (n = 3). 

Fatty acid desaturases were identified in all chromosomes, except for chromosome 10 (Table S14, Figure 6). The highest protein length was noted for PC_05g17080 (*PcDES.1*), a sphingolipid delta (4)-desaturase DES1-like gene with a molecular weight of 58.59 kDa.

**Figure 7:**
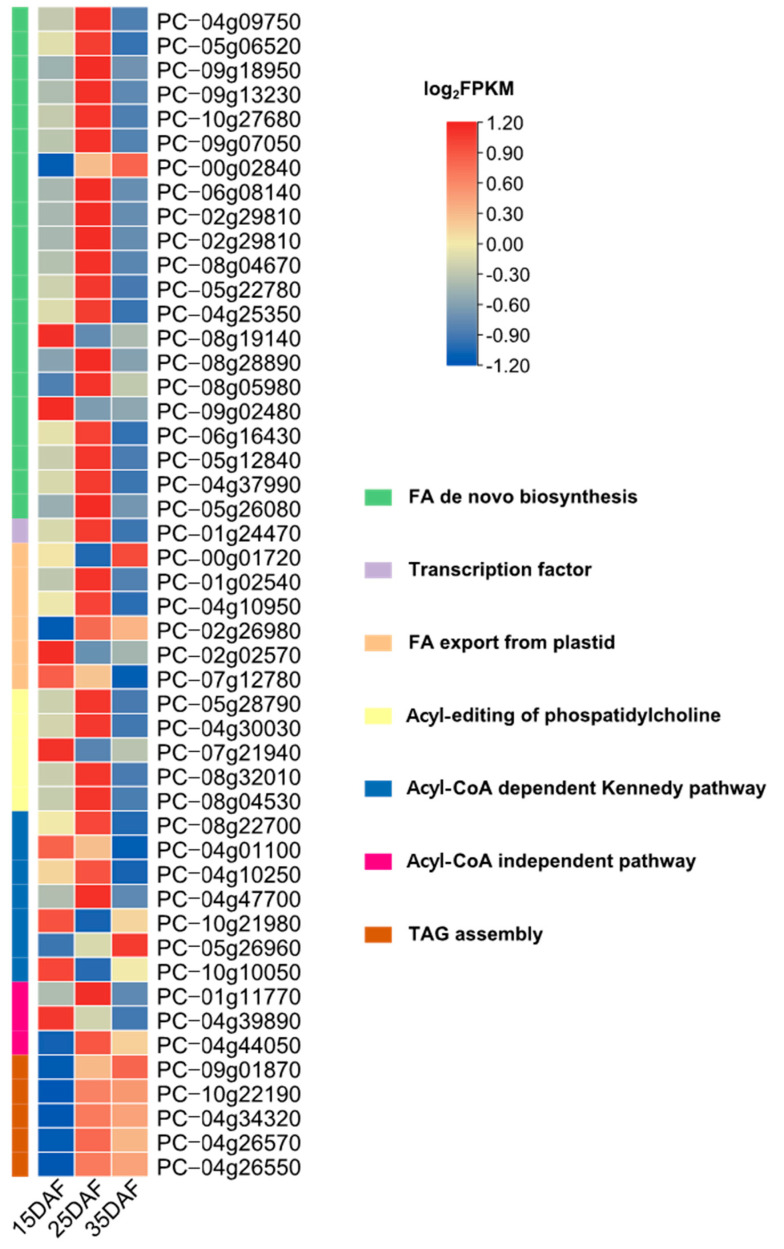
Seed expression of fatty acid and lipid triacylglycerol-related enzymes. A heatmap of the expression dynamics of gene members in seven clusters (FA de novo biosynyhesis, transcription factor, FA export from plastid, acyl editing of phospatidylcholine, acyl-CoA dependent Kennedy pathway, acyl-CoA independent pathway, and TAG assembly) representing specifically expressed genes is shown.

Regarding the expression of genes involved in fatty acid and lipid triacylglycerol biosynthesis, prominent expression was noted 25 days after flowering (Figure 7). Interestingly, TAG-assembly-related genes were highly expressed later on (after 35 days of flowering), which is similar to the lipid triacylglycerol biosynthesis described by Bates et al. [91].

**Figure 8:**
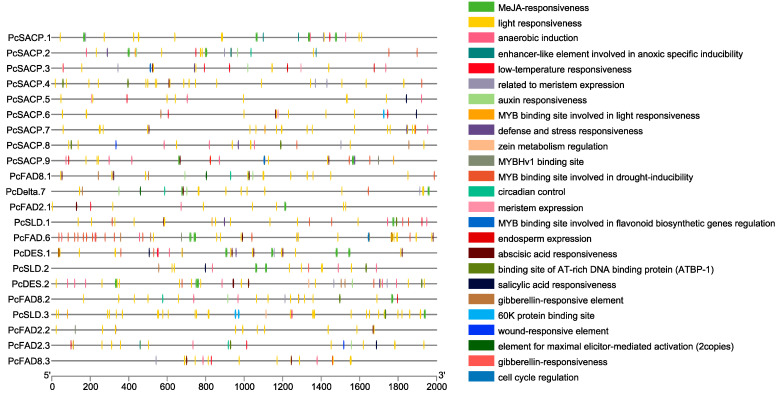
*Cis*-acting element analysis results from a 2 Kbp promoter region of fatty acid desaturases identified in the ‘Jeju17’ genome. The distribution of *cis*-elements in the 2000 bp promoter region related to abiotic stress responses is depicted. Each *cis*-element is represented by a specific color.

*Cis*-acting element analysis in a 2 Kb promoter region of fatty acid desaturase genes was revealed. Some diverse distribution patterns of *cis*-acting elements were observed in the promoter region of fatty acid desaturase genes, indicating that fatty acid desaturase is important in various biological processes. This study identified twenty-five *cis*-acting elements according to their functional annotations, which were related to hormone responses and abiotic stress. Notably, the genes contained *cis*-acting elements related to hormone regulation, such as auxin, gibberellin, methyl jasmonate, abscisic acid, and salicylic acid responsiveness elements. In addition, there is potential implication for the involvement of the genes in diverse biological processes, including light, physiological development, and abiotic stress response (Figure 8). The *Perilla* are closely correlated with lipid metabolism and several metabolic pathways [92].

Fatty acids play important roles in lipid supply in plants and have valuable medicinal properties for humans [93]. This study made a breakthrough in revealing the genetic and molecular determinants of FAs and TAG synthesis in Perilla. Transcriptomics and genomics studies have also disclosed the key enzymes responsible for FAs synthesis in Perilla, including polyunsaturated fatty acid desaturases, acyl-related enzymes, and transcription factors.

## 4. Conclusions

This study provides a high-quality genome resource for the wild *P. citriodora* ‘Jeju17’ that originated from the Korean island of Jeju. Although a genome from a Chinese species was recently released, the genetic specificity of ‘Jeju17’ needs to be explored. Thus, the assembly results were compared with the already published PC002, which is karyotypically *P. citriodora* ‘Jeju17’ and, like ‘PC002’, consists of a diploid species (2n  =  2x  =  20). Alignment of ‘Jeju17’ to the Chinese species ‘PC002’ showed significant collinearity, but sequence inversions, mainly on chromosomes 1, 4, 6, 7, and 10, were also detected. The genomic data enabled the identification of species-specific genes, mainly involved in response to abiotic stress, environment adaptation, metabolite biosynthesis, fatty acids, and lipid biosynthesis. These results suggest that the expression of fatty acid desaturase genes in *P. citriodora* is related to *cis*-elements associated with hormone signal transduction and abiotic stress tolerance. In addition, using a homology-based approach, this study identified a set of 22 fatty acid desaturases potentially involved in the fatty acid production pathway. The present genomic analyses constitute a valuable resource for comparative genomics and *Perilla* breeding for fatty acids and oil-related trait improvement.

## Data Availability

Data are contained within the article or Supplementary Material.

## Supplementary Materials

The following supporting information can be downloaded at: https://www.mdpi.com/article/10.3390/genes14101898/s1, Figure S1. Hybrid assembly strategy using Illumina, PacBio, and Hi-C sequences. #represents the number of scaffold/contig; Figure S2: Whole-genome alignment view of ‘Jeju17’ onto ‘PC002’; Figure S3. Genome evolutionary history. a Divergence times with the node age confidence intervals labeled. b The upper right area (insert) shows the Ks distribution from orthologs between *Perilla citriodora* and each of the eleven species; Figure S4. KEGG enrichment bubble plot depicting the functional attributes of ‘PC002’, ‘PC09’, and ‘PF40’ species-specific gene sets; Table S1: SRA details of the genome and transcriptome data employed in the current study; Table S2: List of species used in the comparative genomics study; Table S3: Alignment results for the BAC clone onto the ‘Jeju17’ genome; Table S4. Conservation of ten BAC-end sequences in scaffolds; Table S5. Summary statistics of genome assemblies of ‘Jeju17’ and ‘PC002’; Table S6: Assembly-based structural variations detected by aligning ‘Jeju17’ onto ‘PC002’; Table S7. ‘Jeju17’ repeats content statistics; Table S8. Predicted non-coding RNA statistics; Table S9. Comparative annotation statistics of ‘Jeju17’ and ‘PC002’; Table S10: KEGG and GO analyses results of the ‘Jeju17’ species-specific genes set; Table S11: KEGG and GO analyses results of the ‘PC002’ species-specific genes set; Table S12: KEGG and GO analyses results of the ‘PC09’ species-specific genes set; Table S13: KEGG and GO analyses results of the ‘PF40’ species-specific genes set; Table S14: Information on the identified fatty acid desaturase genes in ‘Jeju17’.
