# Supplementary material for: The Genome of the Korean Island-Originated Perilla citriodora ‘Jeju17’ Sheds Light on Its Environmental Adaptation and Fatty Acid and Lipid Production Pathways
Source: Genes (Basel). 2023 Sep 30;14(10):1898. doi: 10.3390/genes14101898 (PMC10606934; doi:10.3390/genes14101898)
Supplement: Supplementary file 1 [file genes-14-01898-s001.zip › 5-1_Supplementary_Figure_S1.pdf]

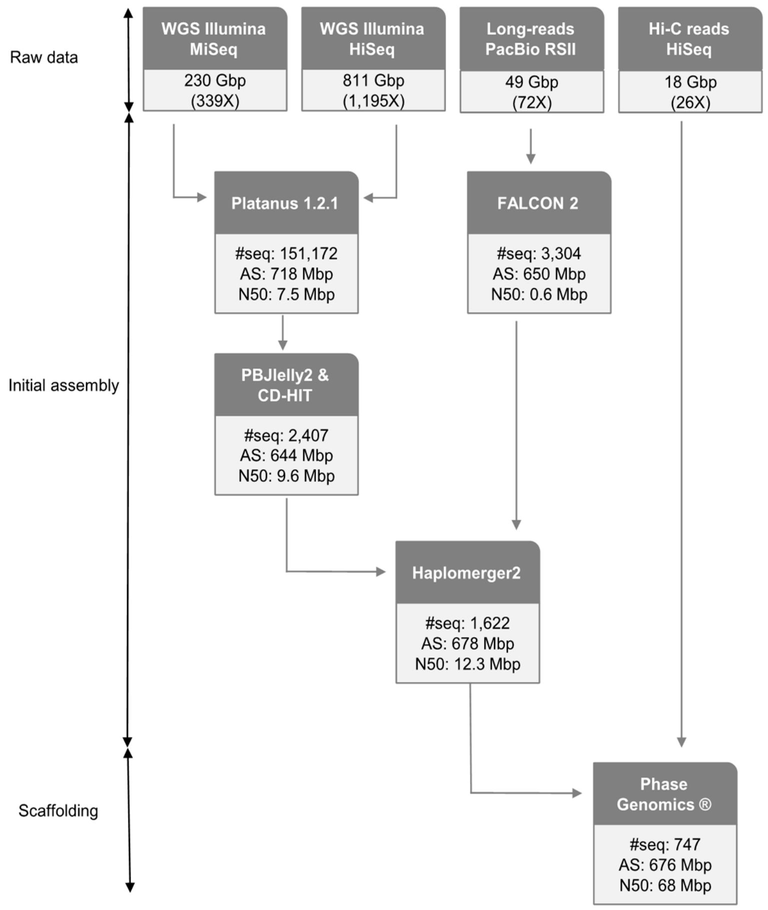

Figure S1. Hybrid assembly strategy using Illumina, PacBio, and Hi-C sequences. #represents the number of scaffold/contig.
