# Supplementary material for: The Genome of the Korean Island-Originated Perilla citriodora ‘Jeju17’ Sheds Light on Its Environmental Adaptation and Fatty Acid and Lipid Production Pathways
Source: Genes (Basel). 2023 Sep 30;14(10):1898. doi: 10.3390/genes14101898 (PMC10606934; doi:10.3390/genes14101898)
Supplement: Supplementary file 1 [file genes-14-01898-s001.zip › 5_3_Supplementary_Figure_S3.pdf]

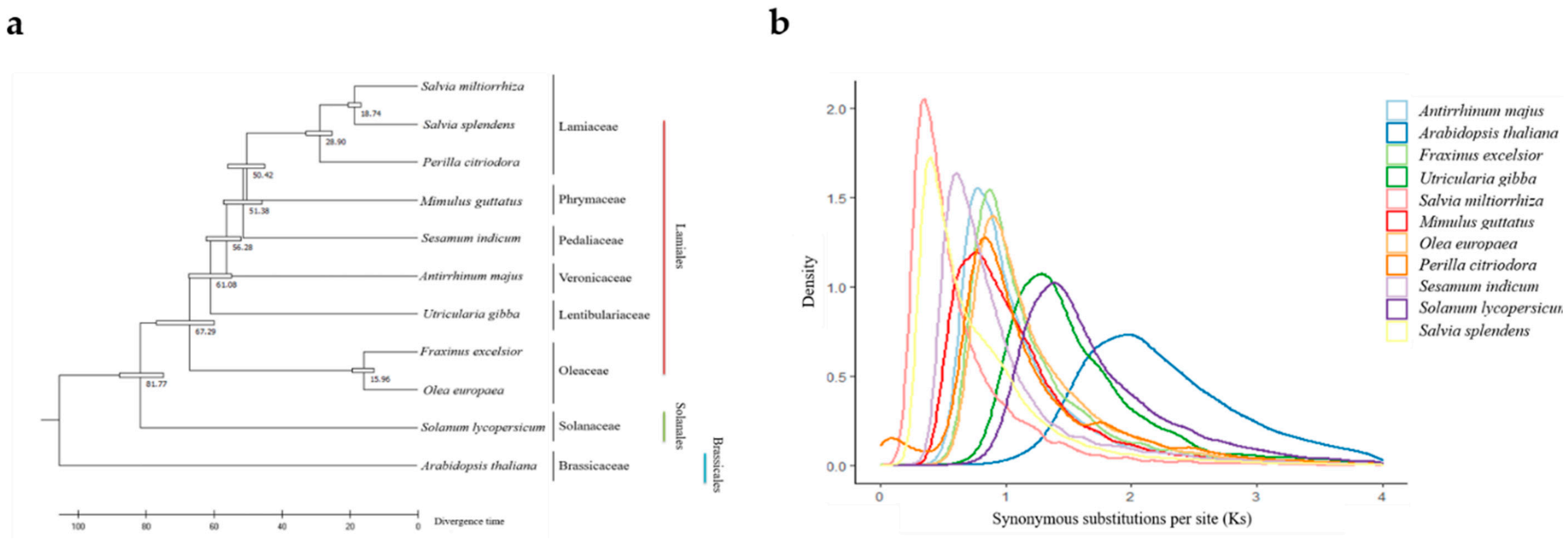

Figure S3. Genome evolutionary history. a Showing divergence times with node age confidence intervals labeled. b Ks distribution on the upper right (insert) is showing Ks distribution from orthologs between *Perilla citriodora* and each of the eleven species.
