# Supplementary material for: The Genome of the Korean Island-Originated Perilla citriodora ‘Jeju17’ Sheds Light on Its Environmental Adaptation and Fatty Acid and Lipid Production Pathways
Source: Genes (Basel). 2023 Sep 30;14(10):1898. doi: 10.3390/genes14101898 (PMC10606934; doi:10.3390/genes14101898)
Supplement: Supplementary file 1 [file genes-14-01898-s001.zip › 5_4_Supplementary_Figure_S4.pdf]

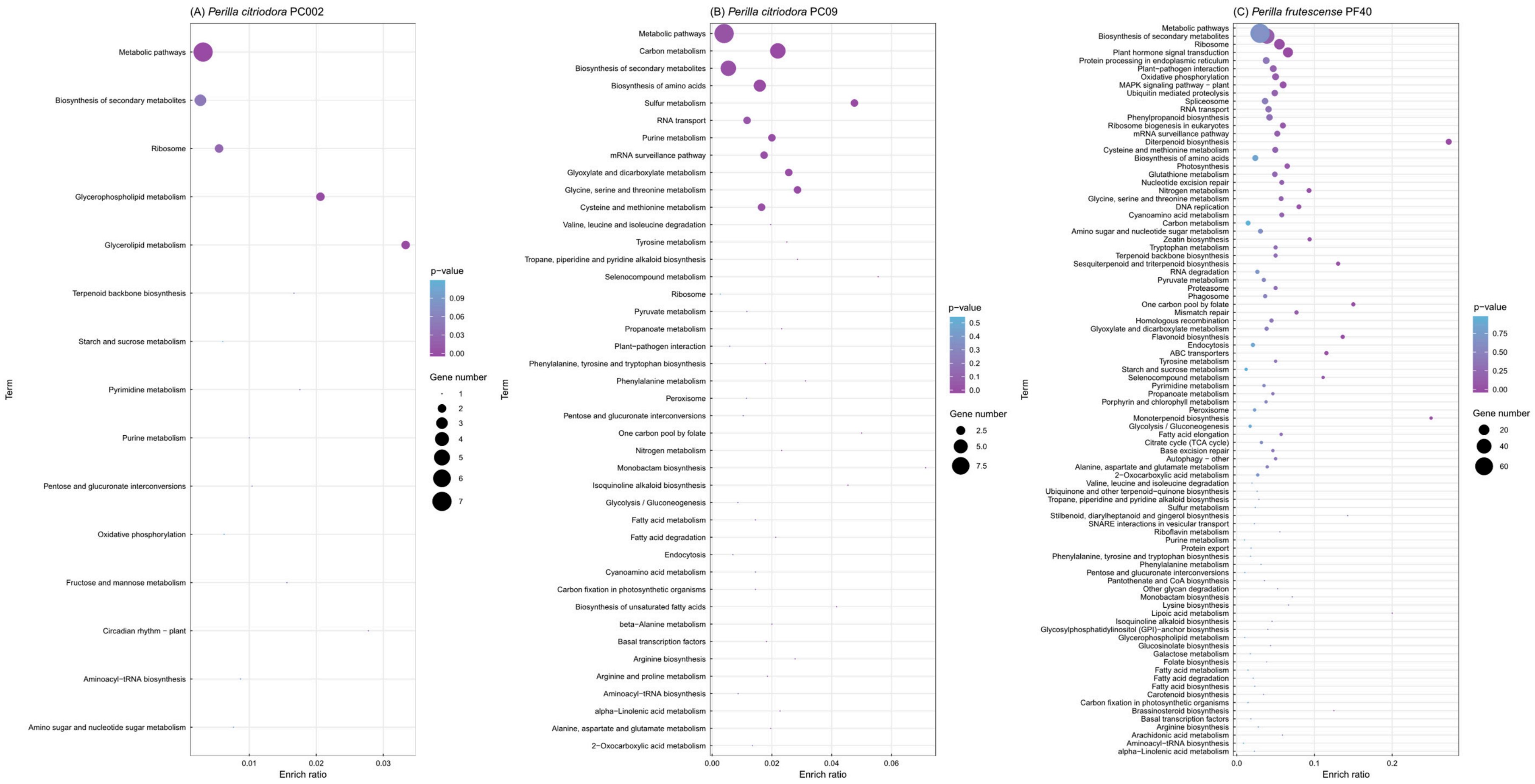

Figure S4. KEGG enrichment bubble plot depicting the functional attributes of 'PC002', 'PC09', and 'PF40' species-specific gene sets.
