# Supplementary figures and images for: The Genome of the Korean Island-Originated Perilla citriodora ‘Jeju17’ Sheds Light on Its Environmental Adaptation and Fatty Acid and Lipid Production Pathways
Source: Genes (Basel). 2023 Sep 30;14(10):1898. doi: 10.3390/genes14101898 (PMC10606934; doi:10.3390/genes14101898)

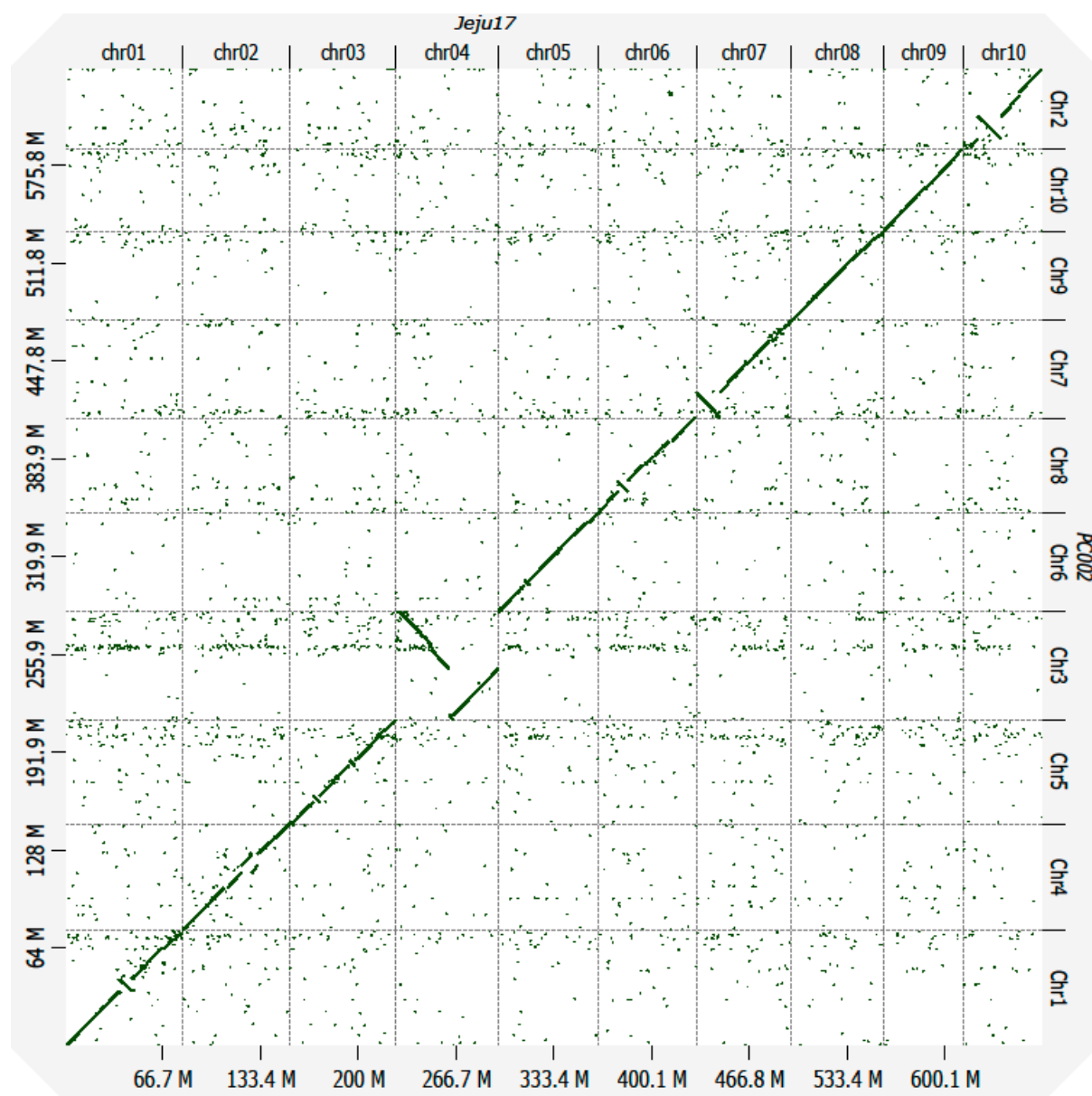

**Figure S2.** Whole-genome alignment view of 'Jeju17' onto 'PC002'.

Supplement: Supplementary file 1 [file genes-14-01898-s001.zip › 5_2_Supplementary_Figure_S2.pdf]
